# Implementation Science to Advance Equity in Climate-Driven Disaster Response

**DOI:** 10.5811/westjem.53809

**Published:** 2026-08-10

**Authors:** William Mundo, Jessica Ryder, Madeline Ross, Corey B. Bills, Lee S. Newman, Jay Lemery

**Affiliations:** * University of Colorado School of Medicine, Department of Emergency Medicine, Aurora, Colorado; † University of Colorado Anschutz Campus, Colorado School of Public Health, Department of Environmental and Occupational Health, Aurora, Colorado; ‡ University of Colorado Anschutz Campus, Colorado School of Public Health, Centers for Health Work and Environment, Aurora, Colorado; § University of Colorado Anschutz Campus, School of Medicine, Department of Medicine, Division of Pulmonary Sciences and Critical Care Medicine, Aurora, Colorado

## Abstract

Climate hazards are causing increasingly frequent and severe disasters. The populations most impacted by the health and social costs of disasters include language minorities, displaced individuals, and low-income communities. This commentary explores three key issues. First, climate hazards lead to displacement and increase vulnerability by disrupting housing, livelihoods, and access to care. Second, current emergency and disaster frameworks often overlook equity in communication, access to care, and recovery. Third, practical solutions can prioritize equity across mitigation, preparedness, response, and recovery. We review evidence on climate displacement, legal protections, and the roles of the health system. We highlight recurring operational opportunities, such as language access, navigation barriers, and fragmented recovery efforts. We also propose feasible strategies aligned with implementation science, including multilingual warning systems, interpreter-ready triage, mobile clinics, community health worker networks, simplified recovery navigation, and policy changes to reduce documentation barriers. This commentary aims to help emergency leaders, public health agencies, and community partners turn intentions into measurable, sustainable equity improvements. Prioritizing language access and legal realities in disaster planning can reduce preventable health issues and accelerate recovery for groups most affected by climate change.

## INTRODUCTION

Climate change acts as a threat multiplier. It accelerates global temperature rise and increases the frequency, severity, and unpredictability of climate-driven hazards with a disproportionate impact on language minority, immigrant, and low-income populations (Table 1).^1^–^7^ Elevated risks of morbidity stem from structural vulnerabilities, such as substandard housing and limited access to health care, often worsened by pre-existing medical conditions, all of which increase exposure to disasters, escalate health impacts, and diminish recovery capacity.^8^,^9^ The Global South and underserved populations in high-income countries face amplified physical health burdens and mental health strains postdisaster, worsened by environmental injustice and weak adaptive infrastructure.^10^–^12^ Without deliberate, equity-centered adaptation, the climate may amplify health inequities and deepen systemic vulnerabilities as extreme weather intensifies.

The human toll of increased frequency and severity of disasters is not shared equally. International standards, such as the United Nations (UN) Guiding Principles on Internal Displacement and the Global Compact for Safe, Orderly, and Regular Migration, affirm the right to protection and nondiscrimination in humanitarian response.^13^ Despite these commitments, many international and domestic disaster management systems lack explicit operational safeguards to ensure equity in communication, shelter access, medical care, and long-term recovery support.^14^–^18^ Existing policies often address vulnerability in general terms but fail to account for the intersecting effects of language, displaced status, and socioeconomic marginalization. This commentary aims to discuss how climate change drives displacement and entrenches vulnerability, identify structural inequities within current disaster systems, and explore scalable, equity-focused innovations for preparedness, response, and recovery.

In this commentary, “language minority” refers to populations whose preferred language differs from that of the dominant social group, including those with limited English proficiency and multilingual communities worldwide. The term “displaced people” includes both refugees who cross international borders and internally displaced persons who remain within their country of origin. Finally, “climate migrants” are those forced to move due to environmental changes that make their home regions uninhabitable or unsafe. While our discussions draw examples from both global and United States (U.S.) contexts, the analysis centers on lessons relevant to emergency medicine, public health, and policy leaders who design or implement disaster response systems. This commentary seeks to inform a cross-disciplinary audience on how implementation science can strengthen equity across all phases of climate-related disaster preparedness and response by bridging humanitarian definitions with operational realities.

## CLIMATE AND DISPLACEMENT

Climate-related disasters are no longer seasonal anomalies but recurring crises that erode community resilience and cause large-scale displacement. According to the Internal Displacement Monitoring Centre, sudden-onset weather events internally displaced more than 26.4 million people within their countries in 2023 alone, and projections from the World Bank suggest that by 2050, over 216 million people could be forced to move within their countries due to climate-related impacts.^19^–^21^ Rising sea levels, for example, are gradually making coastal areas uninhabitable and threatening entire island nations, while persistent droughts, aridity, and intensifying wildfire seasons are displacing millions globally and in the U.S.^22^–^24^ Drought also fuels both rural depopulation and political instability, and extreme storms trigger mass evacuations.^25^,^26^ This sustained and repeated displacement creates prolonged recovery cycles, destabilizes local economies, and undermines long-term adaptation capacity, making climate-linked migration one of the defining humanitarian and policy challenges of this century.

Structural inequities in housing, labor, and access to services often place low-income, at-risk of displacement, and language minority populations in areas most exposed to environmental risk.^12^ For example, dense urban areas often amplify heat exposure via the urban heat island effect.^27^ At the same time, low-lying coastal or floodplain housing is subject to recurring inundation and storm damage.^28^–^30^ These communities are further disadvantaged by limited adaptive capacity, including inadequate insurance coverage, limited financial reserves, and restricted mobility, all of which can prolong postdisaster hardship during both evacuation and recovery.^12^ These patterns reveal that vulnerability is socially produced and geographically reinforced. Building equitable climate adaptation through resilient infrastructure, affordable housing reform, and targeted disaster planning is, therefore, a core public health priority.

**Population Health Research Capsule**

What do we already know about this issue?

*Climate disasters disproportionately harm language minority, displaced, and low-income communities, yet equity remains inconsistently integrated into disaster response*.

What was the research question?

*How can implementation science help build more equitable climate disaster preparedness, response, and recovery?*

What was the major finding of the study?

*PRISM and RE-AIM offer a practical framework to integrate equity into disaster planning and implementation*.

How does this improve population health?

*An implementation science approach may strengthen equitable disaster systems by improving communication, access to care, and recovery for vulnerable populations*.

Displacement disrupts nearly every dimension of health and social stability. It breaks continuity of care, medication access, mental health stability, and social bonds, especially in communities already facing systemic barriers and low trust in institutions. The loss of consistent health care is especially damaging for individuals managing chronic diseases such as diabetes, asthma, and hypertension, conditions that often worsen after disasters.^31^,^32^ The trauma of losing a home or loved one is intensified by secondary trauma from navigating unfamiliar systems, dealing with xenophobia or racism, and confronting restrictive health care policies.^33^–^35^ For children, forced relocation interrupts education and mental health support and destabilizes their development during crucial years, leading to long-term health and psychosocial issues.^36^,^37^ The loss of familiar networks and community supports weakens social cohesion, a critical factor for recovery and resilience.^38^,^39^ Workers exposed to climate extremes, including agricultural and construction laborers, experience heat stress, unsafe conditions, and income loss.^40^ These intersecting disruptions reveal how displacement extends beyond physical dislocation; it fractures systems of care, livelihoods, and trust. Strengthening trauma-informed, culturally responsive health and social services within disaster planning frameworks is essential to protect displaced individuals and rebuild resilient communities.

Climate migrants are individuals forced to relocate because of climate-related events that make their home environments unsafe or uninhabitable.^41^ Policymakers increasingly recognize this population as one of the fastest-growing groups of displaced people worldwide. At the end of 2024, 123.2 million people worldwide had been forcibly displaced by conflict, persecution, or events that disturb public order; of these, 42.7 million were refugees who crossed borders, while 73.5 million were internally displaced persons who remained inside their own countries.^42^ The predominance of internal displacement emphasizes that most displaced individuals travel short distances rather than crossing borders. Because international refugee law applies only to those displaced by persecution or conflict, not environmental change, internally displaced climate migrants often lack the legal recognition and access to international protection given to refugees. These distinctions highlight a critical policy gap that will influence future humanitarian and health equity responses to climate-driven migration.

Currently, there is no binding international legal recognition or protection for climate refugees (Table 2).^43^–^51^ Under existing law, the 1951 Refugee Convention does not cover those displaced solely by environmental factors, leaving externally displaced climate migrants in a legal void.^52^,^53^ Some frameworks, such as the UN Global Compact for Migration, the Sendai Framework for Disaster Risk Reduction, and the Guiding Principles on Internal Displacement, provide soft-law guidance, but they lack enforceable rights and obligations.^54^,^55^ Climate-related disasters are one of the main drivers of internally displaced persons, forcing individuals to move within their own borders in search of safety.^56^ Internally displaced persons often lose homes, livelihoods, and access to care while remaining outside the scope of international refugee protections. The UN High Commissioner for Refugees (UNHCR) climate action framework emphasizes that national governments must strengthen resilience, protect rights, and integrate climate displacement into disaster risk reduction and humanitarian response planning.^57^ Proposed solutions include expanding the definition of refugees to include climate displacement, creating a dedicated climate migration treaty, or embedding protections within human rights frameworks.^58^,^59^ However, these proposals face political resistance, concerns over state sovereignty, and limited consensus on burden-sharing, which hinder progress.^60^ In practice, most protections remain nonbinding, fragmented, and reactive, leaving climate migrants vulnerable to inconsistent treatment, xenophobia, and exclusion from resettlement programs. These gaps underscore the need for a coherent, enforceable global standard that integrates migration policy with climate adaptation and human rights protections.

## HEALTH EQUITY IN EMERGENCY DISASTER RESPONSE: GAPS AND OPPORTUNITIES

Health equity in disaster response means that all people have equal access to timely warnings, emergency care, and recovery resources regardless of language, income, or legal status. Emergency departments (EDs) often serve as a first point of care during crises and are designed to provide acute stabilization. However, sometimes, EDs are also the only point of care for those impacted by climate disasters. When ED care is not followed by longitudinal care or postdisaster follow-up by pre-existing or temporarily activated systems, critical gaps in ongoing health management can arise for affected populations.^61^ International health care systems are increasingly strained by climate-related events such as heat waves, floods, and storms, and many are still working to develop the necessary infrastructure, staffing, and preparedness systems to effectively incorporate climate resilience into their daily operations.^62^ While there are increasing efforts to incorporate focused consideration of vulnerable populations in disaster response planning, refugee and immigrant populations have been historically excluded and face additional barriers to participation, such as fear of immigration and enforcement and lack of trust in health systems.^63^–^65^ Without sustained investment for interpreter services, culturally informed care models, and long-term recovery mechanisms, EDs and disaster systems will continue to fall short of providing equitable care during and after climate-related events.

Disaster response systems face interconnected barriers that operate across communication, policy, and workflow levels. These challenges increase risk for linguistic minorities, undocumented immigrants, and low-income communities, contributing to preventable illness, delayed recovery, and systemic exclusion (Table 3). Emergency communications are often issued only in the dominant language, leaving many unable to receive timely evacuation orders, health advisories, or hazard updates.^66^,^67^ In Australia, mortality rates among people born overseas during disasters were nearly three times higher than for those born locally, mainly due to language barriers and limited English or digital literacy.^68^ Similar issues occur in multilingual regions of the U.S., where residents with limited English proficiency report missing critical alerts or misunderstanding evacuation instructions when messages are not translated or delivered through digital platforms inaccessible to older, rural, or low-income populations.^66^,^69^ Adding to these barriers is a reliance on superficial cultural competency. Training is often viewed as a mere compliance requirement rather than an integral part of implementation that genuinely incorporates multilingual, culturally-tailored communication strategies into disaster response.^70^–^72^

Emergency operations plans and emergency preparedness guides rarely incorporate immigration status, language access, or health literacy into planning, defaulting to a “one-size-fits-all” approach that incompletely accounts for inequities influencing exposure, evacuation, and recovery.^73^ Recovery services, including housing assistance, medical care, and financial aid, are more difficult for non-English speakers to access due to a lack of multilingual navigation support, exacerbating disparities long after the disaster impact.^11^,^74^ Even when cultural competency is included in preparedness frameworks, it remains challenging to operationalize, given the many layers of disaster response.^75^ The result is a persistent inequity, where populations with limited access before a disaster often experience delayed recovery afterward, reinforcing structural vulnerability rather than resilience.

Addressing these inequities requires reframing disaster response as an equity-centered system rather than simply a logistical operation. Disasters reveal, rather than create, the structural weakness of health and social systems. Embedding equity into disaster planning demands more than translation services or temporary outreach. It requires the consideration of the factors underlying who are most affected and why they are vulnerable. This shift helps lay the foundation for innovation that applies implementation science to sustain equitable outcomes across all phases of the disaster cycle.

## INNOVATIONS AND SOLUTIONS: CENTERING EQUITY IN DISASTER RESPONSE

A structured framework is essential to translate equity from principle to practice in disaster response. The Practical, Robust Implementation and Sustainability Model (PRISM) and the Reach, Effectiveness, Adoption, Implementation, Maintenance (RE-AIM) framework together offer a pragmatic, evidence-based approach for designing, evaluating, and sustaining interventions in the real world.^76^,^77^ While PRISM identifies how contextual, organizational, and recipient-level factors shape the feasibility and sustainability of disaster interventions, RE-AIM provides measurable domains for assessing reach, adoption, and long-term impact. Applying these models to disaster planning allows policymakers and practitioners to identify equity gaps early, tailor interventions to local needs, and monitor implementation of integrated strategies across policy reform, community-based response, and technology innovation to advance equitable disaster systems (Figure).

The domains of PRISM could offer a structured approach to align context-specific needs with sustainable interventions, reducing the fragmentation that often undermines disaster response at all stages (Table 4). By mapping environmental, organizational, and recipient factors to each disaster phase, PRISM helps planners anticipate barriers such as funding gaps, workforce shortages, or cultural misalignment before they derail implementation. Integrating this framework with RE-AIM ensures that interventions are both deployable under crisis conditions and evaluated for long-term impact. Policy reforms rooted in PRISM principles can embed equity audits into zoning and funding decisions; community innovations can train and deploy culturally responsive responders; and technology solutions can standardize multilingual communication systems. Together, these approaches demonstrate how implementation science transforms disaster management from reactive aid delivery to a sustained, equity-driven system of preparedness, response, and recovery.

### Policy and System-Level Interventions

Applying PRISM and RE-AIM to disaster policy offers a structured way to operationalize equity across systems that have historically prioritized speed over inclusion while recognizing how sustainability and health equity are critical metrics for program success.^78^,^79^ These frameworks encourage policymakers to analyze how environmental context, organizational readiness, and community engagement influence program sustainability and reach. Embedding this perspective into climate-informed disaster planning enables agencies to map priority populations into preparedness protocols and funding mechanisms. Integrating PRISM and RE-AIM principles can also align policy interventions with existing infrastructure and trusted local networks, supporting equitable scale-up while maintaining feasibility.^77^ Practical applications include expanding emergency health financing and codifying language access requirements into emergency operations plans and drills. Institutional accountability through performance metrics that are tied to equity outcomes ensures these reforms move beyond symbolic commitments toward measurable, system-level change.

### Community-Based Innovations

The PRISM framework has demonstrated value in community health worker and clinical settings where interventions were adapted to local realities while maintaining measurable outcomes.^80^ One illustrative example comes from Haiti’s 2010 cholera outbreak, where a national health agent-led surveillance and education program effectively reduced mortality through community trust and locally embedded training.^81^ Although disease-specific, the model’s success demonstrates how community-trusted intermediaries can strengthen disaster systems in low-resource settings.

Applied to climate-related disasters, this approach involves deploying community health workers trained to identify climate-sensitive hazards, coordinate early response, and deliver culturally and linguistically aligned outreach.^82^ The focus of PRISM on the external environment and recipient engagement provides a framework for tailoring community health worker deployment to community networks, such as churches, shelters, and neighborhoods. Just-in-time responder training, focused on cultural humility, operational stress injury, and bias, could help responders rapidly adapt to the social and cultural context while mitigating and preventing trauma caused by discrimination or miscommunication.^83^–^85^ Crisis navigation hubs that integrate legal aid, health care, and housing services in one-stop centers could address the fragmentation that often undermines recovery for displaced individuals.^86^ These multi-sector models exemplify how implementation frameworks bridge the gap between policy vision and lived community experience.

### Communication and Technology

An implementation science approach can identify contextual barriers such as language, digital access, and trust, which often determine whether communication strategies succeed or fail. By applying these principles, planners can ensure that technology-based interventions are adapted to community capacity and cultural context rather than imposed from the outside. Practical strategies include mobile, multilingual response units that deliver real-time updates and triage support after disasters, and hyper-localized, multilingual warning systems using short message service (SMS), radio, and trusted community messengers. Artificial intelligence-enabled translation and natural language processing tools can enhance accessibility in clinical workflows by generating personalized, easy-to-read discharge summaries in multiple languages or community alerts, thereby embedding language accessibility into digital health solutions.^87^ These solutions must be deployed within transparent governance models for digital health research that aim to enhance data quality and translate research findings into integrated frameworks for planning, executing, and assessing public health initiatives.^88^

Integrating technology with policy reform and community innovation within the PRISM and RE-AIM frameworks provides a unified model for equitable disaster preparedness and response. This model explicitly maps vulnerable populations within preparedness plans, aligns interventions with trusted local infrastructure, and embeds language accessibility into both policy and practice. Deploying multilingual response units, community health workers, and culturally adapted training creates a multi-level approach that connects communication, clinical care, and recovery support. When equity becomes a design requirement rather than a retrospective correction, disaster systems move closer to achieving resilience that includes every community.

## CONCLUSION

Displacement, disconnection from care, and systemic neglect fall heaviest on those individuals without legal protections, linguistic minorities, and economically marginalized communities. Existing disaster management frameworks, designed for speed rather than equity, can leave these populations disproportionately vulnerable to disaster impacts. By aligning community-based solutions, policy reforms, and technology innovations within implementation science frameworks, disaster response can be transformed to incorporate a proactive approach to improve equity. The climate crisis is inseparable from the equity crisis; only by addressing both together can disaster response systems be built to truly safeguard every community when the waters rise.

## Figures and Tables

**Figure:**
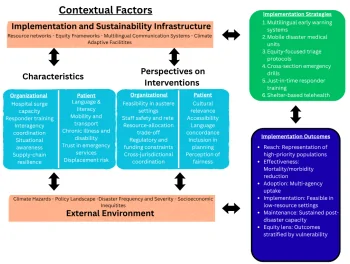
*Conceptual Practical, Robust Implementation and Sustainability Model/Reach, Effectiveness, Adoption, Implementation, Maintenance* framework illustrating integrated policy, community, and technology strategies to advance equity in climate-related disaster preparedness and response. The model links contextual factors, organizational and community perspectives, targeted implementation strategies, and equity-focused outcomes to guide design, scaling, and sustainability across austere settings.

**Table 1:** Climate-driven hazards and impacts.

Hazard	Description	Proximal Impact (Direct or Individual)	Distal Impact (Indirect or Structural)
Sea-level rise	Coastal inundation, freshwater contamination, and salinization of freshwater.	Injuries from flooding, diarrheal disease from water contamination, and mental health disorders from displacement.	Permanent loss of habitable land, displacement of communities with no relocation resources, cultural erasure, long-term livelihood collapse (fishing, coastal farming), and intergenerational trauma.
Extreme weather events	Intensified hurricanes, floods, and wildfires.	Physical trauma (burns, drowning, crush injuries), acute respiratory illness, infectious disease outbreaks, PTSD, and depression exacerbation.	Housing insecurity, economic destabilization, disrupted education systems, and cycles of poverty are tied to recurrent disaster exposure.
Drought and desertification	Persistent water scarcity, soil, and land degradation.	Acute malnutrition, dehydration, diarrheal illness, vector-borne outbreaks, and mental health impacts from food insecurity	Food insecurity and forced migration among subsistence farmers, as well as economic collapse in communities reliant on informal labor, are linked to declines in farming, fishing, and herding.
Environmental degradation	Air quality, drought, aridity, soil erosion, water quality/scarcity, and biodiversity loss.	Heat illness, asthma/COPD exacerbations, cardiovascular events, and mental health disorders are linked to chronic exposures.	Long-term biodiversity-driven nutritional decline and reduced adaptive capacity of health care systems.

*COPD*, chronic obstructive pulmonary disease; *PTSD*, post-traumatic stress disorder.

**Table 2:** Current legal and policy protections for people displaced by climate change.

Legal or Policy Approach	Mechanism of protection	Regulatory body	Strengths	Gaps
1951 Refugee Convention and 1967 Protocol	Defines “refugee” based on persecution; the principle of non-refoulement.	UNHCR / States Parties	Binding, widely ratified international status and protections.	The definition does not cover displacement solely from climate or environmental causes.
UN Guiding Principles on Internal Displacement (1998)	Soft-law human-rights standards for IDPs, including those displaced by disasters.	UN (OCHA), implemented by states	Explicitly applicable to disaster-related IDP; widely used normative guide.	Nonbinding, no enforcement, applies only within a person’s own country.
Kampala Convention (2009)	Regional treaty protecting IDPs due to conflict and natural disasters.	African Union / States Parties	A binding regional law that explicitly covers disaster and climate-related displacement.	Regional (Africa-only)
Paris Agreement - Article 8 & WIM (2015)	Political framework to address loss or damage and displacement, WIM coordinates workstreams and support.	UNFCCC / Conference of the Parties	Formally recognizes displacement associated with climate impacts and provides an institutional home for coordination and finance discussions.	Lacks enforceable individual rights, finance, and operationalization remain limited.
Global Compact for Safe, Orderly and Regular Migration (2018)	Non-binding cooperation framework acknowledging climate and environmental drivers of migration.	UN system (IOM/UN Migration Network)	Recognizes climate as a migration driver, encourages policy coordination and data.	Voluntary creates no legal status or justiciable rights.
Teitiota v. New Zealand (UN, HRC, 2020)	HRC found climate impacts can, in some cases, trigger non-refoulement under ICCPR (right to life).	UN HRC	First UN treaty body view recognizing climate risk may bar removal where life risk is substantial.	Views do not capture other “climate refugee” statuses, are case-by-case, and are non-binding on all states.
G20 LEMM Fortaleza Declaration (2024)	Re-affirms just transition, commits to addressing labor inequities, occupational safety (heat stress), and social protection in the context of climate change.	G20 and Employment Ministers	Global political momentum is linked to climate change, work, a just transition, and worker protections. Endorses the ILO Decent Work Agenda.	Non-binding declaration, relies on national uptake and G20 follow-through.

*HRC*, Human Rights Council; *ICCPR*, Initialism of International Covenant on Civil and Political Rights; *IDP*, internally displaced persons; *ILO*, International Labour Organization; *IOM*, International Organization for Migration; *LEMM*, Labour and Employment Ministers’ Meeting; *OCHA*, Office for the Coordination of Humanitarian Affairs; *UN*, United Nations; *UNFCCC*, United Nations Framework Convention on Climate Change; *UNHCR*, United Nations High Commission for Refugees; *WIM*, Warsaw International Mechanism.

**Table 3:** Multi-level barriers to equitable disaster response and their proximal and distal impacts.

Barrier	Proximal Impact	Distal Impact
Language access gaps	Missed warnings, miscommunication during medical care, and difficulty locating shelters or evacuation routes.	Reinforcement of systemic inequities in disaster communication, erosion of trust in institutions, and widening health disparities among language minority communities.
Fear of immigration enforcement or documentation concerns	Avoidance of shelters and medical care, delayed or forgone treatment during emergencies.	Entrenchment of exclusionary systems that discourage immigrant populations from engaging with disaster infrastructure.
Poverty and resource limitations	Inability to purchase supplies, evacuate, or rebuild, and greater exposure to unsafe conditions.	Cyclical dependency on overstretched aid systems, slower community recovery, and intergenerational loss of wealth and stability.
Preparedness and workforce capacity	Lack of trained interpreters, multilingual responders, and culturally responsive clinicians during crises.	Systemic underinvestment in equitable preparedness infrastructure and limited institutional capacity to adapt to diverse populations in repeated disasters.
Data collection challenges and situational awareness gaps	Lack of recognition of disparities at the individual level, and invisibility of vulnerable populations in immediate disaster statistics.	Persistent gaps in preparedness planning and policy-making, structural neglect in resource allocation, and recovery funding.

*COPD*, chronic obstructive pulmonary disease; *PTSD*, post-traumatic stress disorder.

**Table 4:** Practical, Robust Implementation and Sustainability Model-aligned disaster phase-specific innovations examples.

Disaster Phase	External Environment	Recipients	Design and Adaptation	Infrastructure
Mitigation and Prevention	Long-term policy reforms, land-use planning, and investment in climate projects such as resilient housing and infrastructure.	Linguistic minorities and undocumented communities in high-risk zones.	Inclusion of equity audits in zoning and housing codes, and expansion of flood insurance access, integrated exercises with linguistic minority individuals	Sustainable funding for resilient infrastructure, integration of climate migration into urban planning.
Preparedness	Community risk mapping	CHW outreach to linguistic minority and/or undocumented residents	Multilingual early warning systems	Language-trained hotline staff, mobile alert platforms
Response	Coordination with local and state government agencies, NGOs, and the community	Displaced and medically complex patients	Mobile clinics, pop-up pharmacies	Cross-trained interpreters in care areas, telehealth-enabled triage
Recovery	Policy reform for ID-free aid access	Linguistic minority and low-literacy populations	Simplified aid applications, peer or cultural navigator programs	Sustained funding for CHW networks, legal aid partnerships

*CHW*, community health worker; *ID*, identification; *NGO*, non-governmental agencies.
